# Epidemiology of paediatric renal stone disease: a 22-year single centre experience in the UK

**DOI:** 10.1186/s12882-017-0505-x

**Published:** 2017-04-18

**Authors:** Naomi Issler, Stephanie Dufek, Robert Kleta, Detlef Bockenhauer, Naima Smeulders, William van‘t Hoff

**Affiliations:** 1 Great Ormond Street Hospital for Children NHS Foundation Trust, Great Ormond Street, London, WC1N 3JH UK; 2 Institute of Child Health, University College London, 30 Guilford St, London, WC1N 1EH UK; 3 Centre for Nephrology, University College London, Rowland Hill Street, London, NW3 2PF UK

**Keywords:** Epidemiology, Stone disease, Nephrolithiasis, Hypercalciuria, Growth

## Abstract

**Background:**

Whilst still rare, the incidence of paediatric stone disease is increasing in developed countries and it is important to evaluate the aetiology. We set up a dedicated renal stone service for children combining medical and surgical expertise in 1993 and now have a large case series of children to investigate the epidemiology.

**Methods:**

A retrospective hospital note review of children presenting with kidney stones during the last 22 years (1993–2015) was conducted. All patients had a comprehensive infective and metabolic screen and were classified as metabolic, infective or idiopathic stone disease.

**Results:**

Five hundred eleven patients (322 male) were reviewed. The median age of presentation was 4.4y for males (1 m-16.6y) and 7.3y (1–18.5y) for females with a median height and weight on the 25th centile for male and on 10th and 25th for female, respectively. One hundred seventy five (34%) had an underlying metabolic abnormality, 112 (22%) had infective stones and 224 (44%) were classified as idiopathic.

Of the 175 patients with a metabolic abnormality: 91 (52%) had hypercalciuria (76 persistent and 15 transient), 37 (21%) hyperoxaluria, 38 (22%) cystinuria, 3 (2%) abnormalities in the purine metabolism and the remainder other metabolic abnormalities. Bilateral stones occurred in 27% of the metabolic group compared to 16% in the non-metabolic group (OR 0.2, *p* < 0.05). Urinary tract infection was a common complication (27%) in the metabolic group.

**Conclusions:**

In this paper, we present the largest cohort of paediatric stone disease reported from a developed country giving details on both, clinical and laboratory data. We show that in the majority of the patients there is an identifiable underlying metabolic and/or infective aetiology emphasizing the importance of a full work up to provide adequate treatment and prevent recurrence. Moreover, we show that stone disease in children, in contrast to the adult population, does not seem to be associated with obesity, as children have a weight below average at presentation.

## Background

Whilst still rare, the incidence of paediatric stone disease is increasing in developed countries and is associated with significant morbidity [1–3]. It is especially important in children to understand the epidemiology of stone disease in order to provide adequate treatment and to develop preventive strategies. However, like many paediatric disorders, data on children with stone disease are rare compared to the adult population. In the last British epidemiological study of paediatric nephrolithiasis, in 2003 [4], our group concluded that 44% of stones were principally secondary to a metabolic abnormality, 30% were classified as infective and the rest categorise as idiopathic. Fifty percent of stone composition was calcium-phosphate or calcium oxalate. To examine if these findings are still relevant one decade later, we have repeated this review in stone disease in our centre over the past 22 years.

## Methods

We performed a retrospective note review of radiological and metabolic data for all children presenting during the period from November 1993 to August 2015, including patients previously reported in 2003 by Coward et al. [4]. Since 1993, all children presenting with a renal stone to Great Ormond Street Hospital NHS Foundation Trust (GOSH) receive a comprehensive metabolic evaluation. For retrospective analysis of anonymised patient data no specific ethical approval is needed in our Trust. All children were reviewed by a consultant paediatric nephrologist (WVH) in a dedicated, multidisciplinary paediatric renal stone clinic using a specific list of questions and a standard metabolic screen. All children were screened with plasma levels of urea, creatinine, serum electrolytes, including calcium, phosphorus, magnesium, bicarbonate, alkaline phosphatase, albumin, urate, vitamin D and PTH. As our clinic is hold in the morning and patients usually had breakfast before they travelled to the hospital, samples used for blood and urine analysis were mostly non-fasted samples. Their mid-morning urine was analysed for infection by formal microscopy and culture, and sent for determination of calcium, urate, oxalate, cystine and creatinine (by standard laboratory methods). Urinary citrate was not measured routinely but only at the discretion of the treating physician based on clinical indication.

Where possible, 24-h urine collection was performed. Stones were analysed in a recognised laboratory for chemical composition by IR-spectroscopy.

Medical treatment was obtained retrospectively from the hospital notes. Initially, 543 attenders to the stone clinic were identified in the 22-year period, of which 32 were omitted due to inadequate patient details or having no evidence of stone according to expert radiologist interpretation of the referral patient status.

Patients were classified as having a metabolic predisposition for stone formation according to the following criteria [5]:

### Hypercalciuria

Was defined as a non-fasting, spot urinary calcium: creatinine (Ca:Cr) ratio above the age specific Western society reference values (Birth-1 year up-to 2.2 mmol/mmol; 1–2 years 1.5 mmol/mmol; 2–3 years 1.4 mmol/mmol; 3–5 years 1.1 mmol/mmol; 5–7 years 0.8 mmol/mmol; 7–17 years 0.7 mmol/mmol) [6]. If the child was old enough to cooperate, a 24 h urine was collected (Adequate collection was estimated via measuring 24 h creatinine of 0.1–0.2 mmol/kg/day); hypercalciuria was diagnosed if the calcium excretion was 0.1 mmol/kg/day or above [7]. Minimum of two abnormal results were considered as hypercalciuria. A child who had a raised Ca:Cr ratio that returned to normal after stone removal was defined as transient hypercalciuria. Children with persistent hypercalciuria who were forming stones despite increasing fluid intake and low salt diet, were treated with Thiazide diuretic.

### Hyperoxaluria

Was defined as a non-fasting, spot urinary oxalate: creatinine (Ox:Cr) ratio above the age specific Western society reference values (Birth-1 year up-to 98 μmol/mmol; 1–4 years 72 μmol/mmol; 5–12 years 71 μmol/mmol; above 12 years 38 μmol/mmol) [6]. Abnormalities were confirmed by another spot Ox:Cr ratio or if possible, by a 24 h urine collection into an acidified container [8]. Normal values considered as 100–460 μmol/day/1.73 m^2^ of oxalate excretion. (Adequate collection was estimated via measuring 24 h creatinine of 0.1–0.2 mmol/kg/day) [9]. In cases of persistent hyperoxaluria, urine was also sent for glycolate: creatinine ratio as well as measurement of L-glycerate. Genetic analysis confirmed the diagnosis for primary hyperoxaluria type 1, type 2 or type 3 (AGXT, GRHPR, HOGA1 respectively) [10, 11]. Enteric hyperoxaluria was defined as secondary hyperoxaluria related to congenital gut abnormality, bowel resection or chronic diarrhoea. Patients with primary hyperoxaluria type 1 (PH-1) received a treatment trial of Pyridoxine.

### Cystinuria

Was defined as cystine levels above 20 μmol/mmol creatinine. Other dibasic amino acids (ornithine, lysine, and arginine) were measured to support diagnosis (defined by semi quantitative methods or quantitative urinary amino acid determination by ion exchange chromatography). Children with proven Cystinuria who were forming stones despite increasing fluid intake and alkalinizing urine, were treated with D-penicillamine or Tiopronin.

### Hyperuricosuria

Was defined as repeated increased spot urines of urate: Creatininine level greater than age specific reference values (birth-7 days up-to 1.96 mmol/mmol; 7 days to 2 years 1.53 mmol/mmol; 2–6 years 1.35 mmol/mmol; 6–10 years 0.85 mmol/mmol: 10–18 years 0.67 mmol/mmol) [6]. These were then confirmed with 24 h collections against age specific reference (adequate collection was estimated via measuring 24 h creatinine of 0.1–0.2 mmol/kg/day) [12]. Patients with other purine-containing stones were assessed with more specific metabolic investigations.

### Hypocitraturia

Was defined as citrate levels below 0.11–0.55 mmol/mmol creatinine for females and 0.04–0.33 mmol/mmol creatinine for males.

Patients were classified as having an infective or idiopathic stone formation according to the following criteria: 

Infective. Was defined as the presence of urinary tract infection (UTI) or past medical history of UTI, in the absence of metabolic abnormalities.

Idiopathic. Was defined as the absence of infective and metabolic abnormalities.

### Statistics

Data were analysed for normality using the Kolmogorov-Smirnov test. Non-normal or skewed data are presented in median and range and non parametric tests (or methods) were used for analysis. Significance was assessed by Mann-Whitney *U* Test for non-parametric data and Chi-Square Test for categorical variables. We used IBM SPSS Statistics Version 23 for Mac.

## Results

### Incidence, age, and sex at presentation

**Fig. 1:**
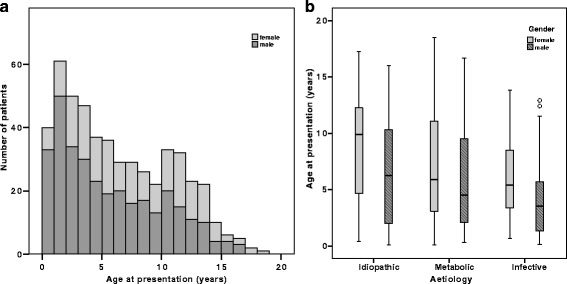
**a** Age of presentation and sex distribution of patients. Male (*n* = 322), Female (*n* = 189). **b** Boxplot showing age of presentation by type of aetiology and stratified by sex

Five hundred and eleven patients (322 boys and 189 girls; 1.7:1 boys: girls), were included over the 22-year period. The median age of presentation was 4.4 years for males (range 1 months - 16.6 years) and 7.25 years (range 1 months - 18.5 years) for females (Fig. 1).

### Presenting features

Most children presented with one or more classic symptoms of stone disease (pain, haematuria, UTI) but the frequency of symptoms was variable (Table 1). Of the total group, only 163 (32%) had a history of abdominal pain, with a lower frequency in children < 6 years, 183 (36%) had UTI, 137 (27%) macroscopic haematuria, and 64 (13%) had painful haematuria (defined as abdominal pain in combination with macroscopic haematuria). In contrast, 67 (13%) patients were asymptomatic and the stone was discovered coincidentally (*n* = 58) or due to positive family history screening (*n* = 9). Seven (1.4%) presented with features of acute kidney injury.

**Table 1:** Presenting features of all patients

Presenting features (*n* = 522)	Number of patients (%)	Missing data (%)
Symptoms	522 (100%)	0
Abdominal pain	163 (32%)	
UTI	183 (36%)	
Macroscopic haematuria	137 (27%)	
Painful haematuria	64 (13%)	
Asymptomatic	67 (13%)	
Coincidental finding	58 (11%)	
Screening	9 (1.8%)	
Acute kidney injury	7 (1.4%)	
Family history	279 (55%)	232 (45%)
positive for stone disease	85 (30%)	
1st degree	31 (11%)	
2nd degree	28 (10%)	
more than 1 relative	26 (9%)	
Premature birth	50 (10%)	0
Immobility	25 (5%)	0
Renal structural abnormalities	93 (18%)	0
Height data	214 (42%)	297 (58%)
Height centile male	25th (0.4–99)	
Height centile female (see Fig. 2)	10th (0.4–91)	
Weight data	209 (41%)	302 (59%)
Weight centile male	25th (0.4–99)	
Weight centile female (see Fig. 2)	25th (0.4–91)	

Features at presentation including the number of missing data where appropriate. Nominal data are presented in n and %; Growth data are presented in median and range

Data on family history were available in 279 patients. Out of those, 85 (30%) had a family history positive for renal stone diseases: first degree relatives in 11%, second degree relatives in 10% and more than 1 relative affected in 9%.

Fifty (10%) children were born prematurely with a median gestational age of 29 (range 24 – 36) weeks. Those children presented significantly younger than the rest of the population at a median age of 2.4 years (range 1 months – 13.3 years) (*p* < 0.05). There was no difference in the stone composition or the family history compared to the rest of the population.

Out of the whole population 25 (5%) had a history of prolonged immobility (mainly wheel-chair bound) secondary to various neurological impairment.

Ninety-three (18%) had primary renal structural abnormalities such as: hydronephrosis, pelvic–ureteric junction stenosis, ureteric-vesico junction stenosis and or cyst. Of those 93 patients, 30 (32%) were defined as metabolic, 21 (23%) infective, and 42 (45%) were idiopathic stone formers.

**Fig. 2:**
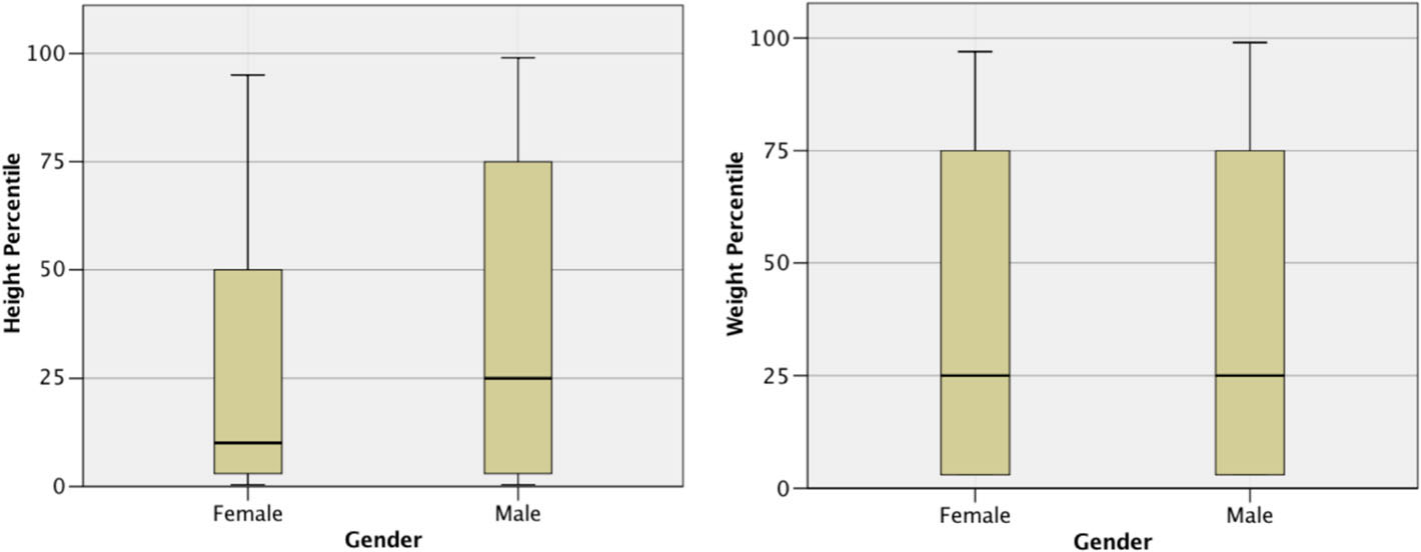
Boxplot of height and weight centile for male and female patients at presentation

Growth data at presentation were available for 214 patients and weight for 209 patients. For the male group the median centile for height was 25th (range 0.4–99) and for weight 25th (range 0.4–99) and for the female group 10th (range 0.4–91) and 25th (range 0.4–91), respectively (Fig. 2). The median centile for BMI at presentation was the 50th (range 3–97) for boys and also the 50th (range 3–95) for girls.

### Aetiology

One hundred and seventy-five (34%) had an underlying metabolic abnormality identified, 112 (22%) were classified as having infective stones and in 224 (44%) we could not identify an aetiological factor. Infective stones were more common among children under 6 years of age, with 70% presenting in this period, whereas metabolic stone formers presented throughout entire childhood.

#### Metabolic aetiologies

Of the 175 patients with a metabolic abnormality: 91 (52%) had hypercalciuria (persistent or transient), 37 (21%) hyperoxaluria and 38 (22%) cystinuria. Three patients (2%) had abnormalities in the purine metabolism (autosomal recessive adenine phosphoribosyl transferase deficiency), 3 patients (2%) had hypercalcemia and 1 patient had hyperuricosuria. In only approximately 10% of the patients urinary citrate was measured, out of which 2 patients had documented hypocitraturia. In the metabolic group 62% were male and the median age of presentation was 5.2 years (range 1 months - 18.5 years) (Fig. 1b). More than a quarter (27%) presented with UTI. Within the 76 patients with persistent hypercalciuria, three (4%) had a diagnosis of Familial hypomagnesaemia with hypercalciuria and nephrocalcinosis [13], one of them previously published [14]. Immobility and prematurity contributed to 16 cases. The remainder had idiopathic hypercalciuria, with no other obvious exacerbating factors such as special diet or medications. In 44 patients from this group we were able to document their family history. Interesting, nearly 40% had positive family history for renal stone. Forty two percent of children having persistent hypercalciuria were treated with Thiazide diuretic (along with strict recommendation to limit dietary sodium intake) trying to increase proximal tubule calcium reabsorption [15].

The hyperoxaluria group included 19 (51.4%) patients with primary hyperoxaluria: twelve with type 1, two with type 2 and five with type 3. In eleven children (29.7%) we could not identify a mutation in one of the known genes and therefore they were categorised as non-type 1–3. The remaining seven patients had enteral hyperoxaluria. Nine patients with PH-1 were treated with Pyridoxine which may stabilise the defective alanine glyoxylate aminotransferase (AGT) enzyme or may act as a chaperone to restore a low level of normal AGT activity [16].

Autosomal recessive Adenine phosphoribosyl transferase deficiency (APRT) was found in 3 patients after stone analysis showed 2, 8-dihydroxadenine [17]. Two presented with UTI and one with abdominal pain and microscopic haematuria.

#### Infective stones

These occurred in 112 patients, median age of presentation was 4.0 years (range 2 months - 13.8 years) (Fig. 1b), the majority were male (68%) and boys were significantly younger at presentation than girls (42.5 vs 65 months, *p* = 0.001). A third (35%) had staghorn stones resulting in acute kidney injury at presentation in one patient. Ten children (9%) were found to have chronic kidney disease (GFR < 90 ml/min/1.73 m2) at the end of follow-up compared to 9 (4%) in the idiopathic group and 16 (9%) in the metabolic group. Children with infective stones presented at a significantly younger age compared to the metabolic group (4 years (range 2 months to 13.8 years) versus 5.2 years (range 1 month to 18.5 years); *p* = 0.016). The male:female ratio was 2.1:1, compared to the metabolic group of 1.6:1 (*p* = 0.336).

### Stone distribution

The stone was in the upper tract in 413 children (81%), unilateral on the left in 180 (44%), on the right in 134 (32%), and bilateral in 99 (24%). In 26 (5%) cases the stones were located in the bladder and upper tract. Metabolic abnormality was found in 47% of children with bilateral stone. The risk of presenting with bilateral stones was increased significantly in children with an underlying metabolic abnormality, with 47 of 175 (27%) having bilateral stones in the metabolic group compared to 52 of 336 (16%) in the non-metabolic group (Odds ratio 2.0; *p* = 0.002). Lower distribution of stone, e.g.,: ureter, bladder or urethra was significantly lower (30%) compared to the presence of stone within the kidney parenchyma or the intrarenal collecting system.

### Stone composition

Stone analysis was available in 300 patients (58%). The majority of stone analysed were composed of calcium oxalate or calcium phosphate (78%). Triple phosphate stones were detected in 14%, and cystine stone in 7%. See Table 2.

**Table 2:** Stone composition and corresponding aetiologies

Stone composition (*n* = 300)	Metabolic	Infective	Idiopathic
Calcium oxalate	44 (14.7%)	8 (2.6%)	50 (16.6%)
Calcium phosphate	42 (14%)	49 (16.3%)	42 (14%)
Triple phosphate	2 (0.7%)	32 (10.7%)	6 (2%)
Cystine	20 (6.7%)	0	0
Uric Acid	0	1 (0.3%)	4 (1.3%)

In 211 cases, stone composition could not be evaluated due to spontaneously passage of gravel or post lithotripsy procedure.

### Sub analysis -comparison 1993–2001 and 2002–2015

In sub-analysis we compared findings of 133 patients from 1993 to 2001, of which 106 were represented also in our first audit [4] with the 378 patients in the more recent era (2002–2015). There was no significant difference in gender, age of presentation and aetiology.

## Discussion

We here present the largest cohort of paediatric stone disease giving details on both, clinical and laboratory data, in western developed countries reported to date [1, 2, 18–25]. This unique study provides data regarding the epidemiology and laboratory characteristics of 511 paediatric kidney stone formers and assessed the aetiology of nephrolithiasis within this distinctive group.

The only paediatric study involving more children although provided only data on microscopic examination and infrared spectroscopy of calculi and no clinical data [26].

The well-documented male preponderance, especially in the younger age group, has again been confirmed [19, 20, 27], although a few paediatric series have shown female predominance [22, 28]. Most epidemiologic studies of symptomatic urolithiasis in adults also show a male preponderance [29, 30].

Most paediatric series note a significant number of adolescents with stones, and an increase in stones in patients in this age group over time [1, 3]. Yet in our series there were few patients who presented after 13 years of age, which may reflect a referral bias in our population. It is possible, that older children may be referred to an adult nephrologist or urologist.

Presentation of nephrolithiasis in children is frequently atypical [19], which may explain why only 32% were perceived to have pain and 13% to have had painful macro-haematuria. Adult patients are more likely to present with the classic signs and symptoms of urolithiasis, such as flank or abdominal pain and gross haematuria [31]. This symptom may go unrecognised in younger paediatric patients, who may find it difficult to localise or describe their symptoms. Urinary tract infection was the presenting symptom in more than a quarter of patients who were subsequently noted as having an underlying metabolic abnormality. This indicates the importance of metabolic evaluation in every child presenting with urinary tract infection and stone disease, primarily to initiate a preventive therapy.

In the adult population obesity has been found to be a risk factor for kidney stone formation [32–34]. Similar findings have been reported in an American study of mainly adolescent stone formers (average age 12 years), where 31% of the children were considered obese [19]. That is in contrast to our findings, where we observed that the median centile of weight for male and female stone formers was below the 50th centile for a healthy population. Moreover, the height was also below the 50th centile for a healthy population, demonstrating that paediatric stone formers are more likely to suffer from failure to thrive or that co-morbidity contributed to growth delay. Dwyer et al. [3] and Sas D.J. [2] reported similar findings, stating that obesity is not a contributing factor to the increasing incidence of kidney stones in paediatric stone formers and in fact they have a lower BMI on average than general paediatric population.

The aetiology of paediatric urolithiasis is influenced by the scope of the metabolic work-up, the accepted definition and by the geographic area. The aim of conducting a metabolic and infective evaluation is to be able to implement an appropriate treatment which may minimise the risk of recurrent stones. More than half (56%) of our patients were found to have metabolic or infective stones, which also prevail in most series throughout the world [1, 20, 22, 28]. Hypercalciuria is the most common metabolic cause of paediatric urolithiasis [35]. It was noted in 43% of our patients with metabolic urolithiasis (15% among the whole cohort). Other groups have found hypercalciuria among patients with urolithiasis to vary from 7 to 34% [20, 22, 28, 36]. Of 37 children with hyperoxaluria, 19 (51%) had primary hereditary variant. The high proportion of such patients in our series reflects the tertiary referral nature of the practice at GOSH. Hypocitraturia is in other studies reported with an incidence up to 15–50% [2, 19]. In our population urinary citrate was unfortunately only measured in selected cases, the decision residing with the treating clinician. Therefore, we cannot draw any epidemiological conclusions regarding the role of hypocitraturia in our cohort.

Infectious stones were found in 22% of patients, 70% were younger than 6 years at presentation. In the vast majority of the infected stone Proteus was isolated. This is consistent with struvite (i.e., ammonium-magnesium phosphate or triple phosphate) urolithiasis occuring in the presence of infection by urease producing microorganisms. In our cohort 32 (29%) of children with infective stone had pure triple phosphate stone, while the rest had calcium-phosphate or calcium-oxalate stone composition. Nearly ten percent from the infective group ended up with some degree of chronic kidney disease (CKD). However, out of all children who ended up with a degree of chronic kidney disease (*n* = 35), 26% had an underlying structural abnormality, the likely main contributor to the development of infection and CKD in paediatric stone formers. Other groups have reported the increased risk of CKD with nephrolithiasis among children and adults, especially in the situation of recurrent urinary tract infections [24, 28, 37]. Further studies are needed to better define and characterise infective stone associated CKD and the patients at increased risk for this complication.

Within idiopathic stone formers 42 (19%) were found to have primary renal structural abnormalities, which may promote urine stasis and increase the risk of calculus formation without any other underlying disease [38, 39]. Thirty-eight (17%) of idiopathic stone formers were born premature and/or had a history of prolonged immobilisation, which can both provide a known risk factor for nephrolithiasis [40, 41].

In our population we could not observe a change in aetiologies over time (1993–2001 vs 2002–2015). Comparing our results to the previous published paper by Coward et al. it appears that the proportion of idiopathic stone formers is increasing with less metabolic and infective aetiology [4]. However, the main reason for this discrepancy is, that in our study more rigorous criteria for classification of a metabolic and infective aetiology were applied. In order to be classified as metabolic abnormal urinary results had to be documented at least twice. Further, to be classified as infective aetiology, a positive urine culture had to be available in our laboratory. Applying these criteria, both to the initial cohort (1993–2001) and the subsequent cohort (2002–2015) we do not see any significant difference (data not shown).

The stone composition is critical for introducing targeted therapy after urinalyses is completed. Like most stone-forming children, our population demonstrates predominantly calcium oxalate and calcium phosphate stones [20, 22, 28, 36]. Comparable to Milliners’ group [22], magnesium ammonium phosphate stones were most often noted in the under 6 years age-group.

Interestingly, bilateral stone disease seems to predict having a metabolic abnormality (48% of our cases). It may also predict a more severe stone disease which demands targeted medical and intervention treatment.

The primary limitation of our study is that it is a retrospective audit, resulting in an absence of uniform data sets. Therefore, we are not able to provide data on crystalluria and morphologic analysis which has been shown to be of interest for the etiologic diagnosis of stone disease without available stone. Secondary, urinary citrate was not measured routinely but only at the discretion of the treating physician. Therefore, our study is not able to reflect the true number of patients with hypocitraturia and we cannot draw epidemiological conclusions regarding this metabolic disorder. Also, comprehensive genetic studies will be needed to define how many patients have a genetic cause of their kidney stone disease.

Furthermore, our results are based on data obtained from patients seen in our clinic and not from a population-based study. Therefore, our results might vary from the overall paediatric stone disease population in the UK.

## Conclusion

In conclusion, we here present the largest cohort of paediatric stone disease in western developed countries published up to date demonstrating that metabolic risk factors accounting for one-third of all calculi with an increased risk of bilateral disease. In contrast to the adult population, obesity is not a risk factor for stone development in the paediatric population.

Future work: Nearly 30% of our children had a positive family history for stone disease, which may indicate a genetic component to urolithiasis [42]. Milliner and his group [22] found even higher numbers particularly among the hypercalciuric subgroup. At present we do not know how many patients have a likely genetic cause of their kidney stone disease. Establishing comprehensive genetic testing for known disease genes is one of our current priorities. We also believe that conducting a genome-wide association study (GWAS) might reveal unknown hereditary traits linked to this phenotype.
